# A Cross-Sectional Study of Mental Health, Lifestyle, and Anxiety in Japanese Nursing Students by Year of College

**DOI:** 10.3390/healthcare10101870

**Published:** 2022-09-25

**Authors:** Kisaki Kobayashi, Etsuko Tadaka

**Affiliations:** 1 Department of Community Health Nursing, Graduate School of Nursing, Sapporo City University, Sapporo 060-0004, Japan; 2 Department of Community and Public Health Nursing, Faculty of Medicine, Graduate School of Health Sciences, Hokkaido University, Sapporo 060-0812, Japan

**Keywords:** nursing students, mental health, lifestyle, college life anxiety factor, grade level

## Abstract

For students in nursing colleges to develop necessary competencies as health care professionals and prevent post-graduation “reality shock” deterioration in the quality of care they provide, appropriate measures are needed in response to changes that occur as college years progress. In this study, mental health status, lifestyle, and various types of anxiety were investigated in 448 nursing college students during a four-year program in Japan. Students from each year of the program were surveyed with the K10 scale to measure mental health, and the Student Life Questionnaire to assess dietary habits, sleep duration, and exercise. Mean K10 scores for first-year students was 13.1, with 30% having anxiety about college life. Similarly, for second-year students the K10 score was 13.7, and compared to first-year students, rates of meal skipping and sleeping less increased. For third-year students, the K10 score (15.9) was higher than for other groups, as were their percentages of all items. Fourth-year students had a mean K10 score of 14.6, with a higher rate of meal skipping and feeling anxious about the future than other groups. These results suggest the need for support tailored to the characteristics of each year in the bachelor’s program in nursing.

## 1. Introduction

While mental health is a universal issue for all people working toward the Sustainable Development Goals [1], one of the most important populations in which to address mental health is adolescents, who are the future generation [2,3]. Suicide is the leading cause of death among Japanese aged 15 to 29 years and is related to environmental factors such as work, college life, and interpersonal relationships [4]. For nursing students in the adolescent age group, who will become important professionals responsible for improving the mental health of the nation in the future, improving their own mental health is a precondition. Previous studies have shown that nursing students have poorer mental health compared to college students in other programs [5,6], which has a significant impact on leave of absence and withdrawal from school [7]. Furthermore, following graduation, nursing professionals have a higher prevalence of depression and anxiety compared to other health care professions [8], and early turnover [9] has become a problem. In order to address or prevent these issues, it is imperative to develop measures that are in line with the actual state of mental health of nursing students today.

A previous study suggested that nursing students with poor mental health are more likely to develop physical symptoms, anxiety, insomnia, social dysfunction, and depression [10]. In addition, the quality of sleep among college students is strongly associated with psychological distress [11], and its impact on lifestyle includes decreased physical activity [12] and lack of active engagement in prevention of health risk behaviors [13]. Furthermore, it has been reported that nursing students perceived clinical stressors more intensely than academic and external stressors [14]. Therefore, in order to maintain and promote the mental and physical health of nursing students from the perspective of lifelong career development, it is necessary to appropriately assess the actual states of their mental health, lifestyle, and anxiety by year of college, and to accumulate data on effective educational methods that are in line with those states. Efforts in this area are not yet sufficient and, in particular, little is known about the characteristics of nursing students’ mental health, lifestyle, and anxiety according to their year of college.

The health care environment in Japan has changed dramatically along with social and demographic trends such as declining birthrate and an aging society, advances of women in society, and the trend toward late marriages and late childbearing [15]. It has also been affected by increasing sophistication and complexity of medical care and advances in medical technology. In recent years, technological innovations such as artificial intelligence, Big Data, and the internet have advanced, and expectations for the training of nursing professionals (i.e., public health nurses, midwives, and nurses) who play a role in health care have increased in both quality and quantity. This has led to a remarkable increase in the number of nursing colleges, from 11 in 1991 to 295 in 2021 [16], accounting for about one third of all 779 colleges in Japan [17]. In order for nursing students to develop the necessary competencies for health care professionals during college, and to prevent the so-called “reality shock” that occurs after graduation when the gap between ideal-ism and reality causes mental health difficulties and consequent deterioration in the quality of health care, it is necessary from the perspective of lifelong learning to pay appropriate attention to the mental health of students as they progress through the develop-mental stages of their four-year bachelor’s degree program.

The current study aimed to investigate characteristics of the mental health, various types of anxiety, and lifestyles of students during each of their four years of nursing college in Japan in order to gain insight into future education for these health care professionals.

## 2. Materials and Methods

### 2.1. Subjects and Methods

Study subjects were 448 students enrolled in a standard four-year nursing college in Hokkaido, Japan: 129 were first-year (21 men, 108 women), 104 were second-year (12 men, 92 women), 104 were third-year (11 men, 93 women), and 111 were fourth-year (13 men, 98 women) students. The study utilized an anonymous self-administered survey.

This study was conducted using a self-administered questionnaire survey on the same day in April 2018 for all research subjects in grades 1 through 4. In order to guarantee free will in the responses, no reminders were given to research subjects who did not respond.

### 2.2. Survey Contents

The K10 scale [18] was used to measure mental health. Reliability, validity, and effectiveness of this tool have been demonstrated in Japan [19]. The questionnaire consisted of 10 questions regarding the past 30 days, such as “Did you feel exhausted for no reason?”, answered on a 5-point scale composed of “1 = none of the time”, “2 = a little of the time”, “3 = some of the time”, “4 = most of the time”, and “5 = all of the time”. The minimum total score was 10 points, and the maximum total score was 50 points.

Lifestyle assessment was based on the Student Life Questionnaire in Japan [20], which included the following four items: dietary habits (whether they are eating regularly or whether they are skipping meals), sleep duration (less than six hours or six hours or more a night), and exercise (whether or not they exercise regularly).

The same Student Life Questionnaire was also used to determine the presence or absence of anxiety in each of five areas: overall studies, clinical practice, future career path, life in general, and personal relationships.

### 2.3. Ethical Considerations

The study was approved by the Ethics Committee of Sapporo University of Health Sciences (IRB no: 017009). Ethical considerations included informing the subjects of the purpose of the research, protecting their privacy through anonymous responses, freedom of response, not being disadvantaged by responses, and anonymity at the time of data release. Submission of the questionnaire on the day of the study was regarded as consent to the survey.

### 2.4. Statistical Analysis

Data analysis was performed using SPSS for Windows (Statistical Package Social Sciences version 26). Descriptive statistics were performed on the respondents’ demographics. One-way analysis of variance (ANOVA) was used to compare the mean K10 scores of the four year groups, and multiple comparisons using Tukey’s HSD were used for comparisons between each group. Chi-square tests were conducted to detect group differences for the three lifestyle variables and five anxiety item variables, and z-tests and Bonferroni method were used for comparisons between year groups.

### 2.5. Proofreading and Native Checking of Manuscript

This manuscript was proofread and native-checked by the National Center for Global Health and Medicine (https://www.nai.co.jp/en/ (accessed on 20 September 2022).

## 3. Results

### 3.1. Respondent Demographics

**Figure 1:**
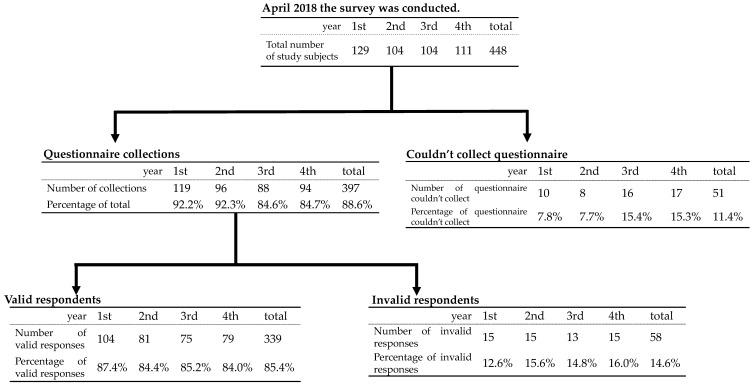
Number and Percentage of Respondents and Number and Percentage of Valid Respondents.

From a total of 448 students, 397 responses were received (88.6% response rate). Among the 397 responses, 339 (85.4%) were considered valid and 58 (14.6%) blank responses were considered invalid. The number of valid responses included 104 first-year students (87.4%), 81 second-year students (84.4%), 75 third-year students (85.2%), and 79 fourth-year students (84.0%) (Figure 1). The number of valid responses by gender was 47 males (13.9%) and 292 females (86.1%), but differences between sexes were not included in the analysis at this time, as it was determined that the sample sizes were too different to make comparisons. Regarding anxiety, 226 (67.7%) students were anxious about their studies, 223 66.8%) about their clinical practice, and 235 (70.4%) about their future, while 243 (72.8%) students were not anxious about their college life, interpersonal relationships (239 [71.6%]), or their personality (226 [65.3%]) (Table 1). In terms of lifestyle, 246 (73.7%) of the 448 students were not skipping meals, 248 (74.5%) were sleeping for six hours or more, and 281 (85.2%) were not exercising (Table 2).

### 3.2. Characteristics of Year Groups

#### 3.2.1. First-Year Students

The mean K10 score for the first-year students was 13.1. One-way ANOVA was conducted to examine group differences in the mean K10 scores, with year group as the independent variable and K10 total score as the dependent variable. Table 1 shows mean scores for the groups, with significant differences seen between groups (F [3, 330] = 2.739, *p* < 0.05). Mul-tiple comparisons using Tukey’s HSD (at the 5% level) showed that K10 scores of first-year students were significantly different from those of third-year students (*p* < 0.05). Regarding anxiety, 223 (66.8%) students reported that they were anxious about clinical practice, compared to 66 (64.7%) of the first-year students who reported that they were not anxious about clinical practice (*p* < 0.001). When comparing year groups, there was a significant difference between the first-year group and the other groups. Regarding lifestyles, there was a significant difference between first-year students and third- and fourth-year students in dietary habits (Table 2). A significant difference was observed between first-year and third-year students with regard to sleep, and 83% of students were not exercising.

#### 3.2.2. Second-Year Students

The mean K10 score of second-year students was 13.8 (Table 1). The percentage of second-year students who were “anxious about studies” was 66.3%, which was higher than the 58.8% reported by first-year students. The percentage of second-year students who were “anxious about clinical practice” was 71.3%, twice as high as that of first-year students (35.3%). The percentage of second-year students who were “anxious about college life” (27 students, 33.8%) and “anxious about interpersonal relationships” (29 students, 36.3%) were the highest compared to other groups. Regarding anxiety about clinical practice, 35.3% of first-year students were anxious, which was about half that of the 71.3% of second-year students. Multiple comparisons revealed a significant difference between second-year students and first- and third-year students (*p* < 0.001) (Table 1). The rate of meal skipping was 26.3%, which was twice that reported by first-year students (11.8%) (Table 2). The number of students who were sleeping less than six hours also increased, from 20.8% in first-year students to 22.5% in second-year students.

#### 3.2.3. Third-Year Students

The mean K10 score of third-year students was 15.9 (Table 1). Multiple comparisons using Tukey’s HSD (at the 5% level) showed a significant difference between third-year and first-year students (*p* < 0.05), with third-year students scoring highest among all four year groups. Third-year students had the highest percentage of anxiety about their studies (66 students, 89.2%) and clinical practice (67 students, 90.5%) compared to the other groups. Specific comparisons showed that a higher percentage of third-year students (66 students, 89.2%) were anxious about their studies than were first-year (60 students, 58.8%), second-year (53 students, 66.3%), and fourth-year (47 students, 60.3%) students (*p* < 0.001). There also were more third-year students anxious about clinical practice compared to first- and second-year students. Regarding lifestyle habits, 31 (41.9%) students were sleeping less than six hours, and 73 (98.6%) students were not exercising, which were the two habits with highest percentages compared to other groups (Table 2). Multiple comparisons demonstrated that the percentage of students sleeping less than six hours was higher compared to first- and fourth-year students. The overall percentage of students exercising was small to begin with, but was significantly (*p* < 0.01) lower among third-year students (73 students, 98.6%) compared to first-year (83 students, 83.0%), second-year (64 students, 81.0%), and fourth-year (61 students, 79.2%) students.

#### 3.2.4. Fourth-Year Students

The mean K10 score of the fourth-year students was 14.6 (Table 1). For feelings of anxiety, 62 fourth-year students (79.5%) were most anxious about the future, compared to other groups. There were significant differences between the percentages of fourth-year and first-year students who were anxious about their clinical practice (*p* < 0.001) and also about their future (*p* < 0.05). Regarding lifestyles, meals were being skipped by 31 (39.7%) students (Table 2), and the percentage of students skipping meals increased as they progressed through the school year, with a higher percentage of fourth-year students skipping meals compared to other groups. Regarding sleep duration, there was no significant difference between fourth-year students and first- and second-year students.

## 4. Discussion

### 4.1. Respondent Demographics

The collection rate was 88.6%. This is a reasonable collection rate for this study, as collection rates in other studies of nursing students have been in the 60% to 80% range [6,13,14].

### 4.2. Characteristics of Year Groups

#### 4.2.1. First-Year Students

The mean K10 score for first-year students (13.1) was lowest among all groups, suggesting that first-year students may have a more stable mental health status. This is possibly because they are excited about being accepted into the first year of college and starting new lives, and because of the greater emphasis placed on a liberal arts curriculum during the first year. Although first-year students were less anxious about studies and clinical practice than other year groups, 30% were anxious about college life, which was the second-highest percentage after the second-year students. As first-year students begin their transition to a new life, they are becoming independent of their parents and are responsible for managing their food, clothing, and living arrangements. It has been suggested that strategies to increase new nursing students’ confidence in interpersonal relationships and studies should be implemented to facilitate their adjustment to college life [7]. To this end, the first thing needed would be “building a foundation for college life”.

On the other hand, many first-year students had a favorable lifestyle in terms of both dietary habits and sleep, although many of them were not exercising. Previous studies have shown that many students in the lower years of college do not exercise [12,21]. As college life progresses, the habit of not exercising may increase body mass index, and in particular, students living alone or in student dormitories change their habits significantly as the school year progresses [22]. Therefore, measures for first-year students should aim at “maintaining a healthy lifestyle” as described in the “More active people for a healthier world” initiative recommended by WHO [23], and the development of lifestyle habits specific to nursing students [24].

#### 4.2.2. Second-Year Students

The mean K10 score of second-year students (13.8) was second lowest among the four year groups, after first-year students.

Second-year students’ anxiety was in line with the results of a previous study [14], with increased anxiety about studies and clinical practice compared to first-year students. This may be because of the transition from a liberal arts curriculum for first-year students to a professional education curriculum for second-year students, which increases their ability to formulate more specific ideas about nursing, and because of the influence of their first on-the-job clinical practice experience. More second-year students were also anxious about their college life and interpersonal relationships compared to other year groups. Second-year students transitioning from adolescence to adulthood (roughly at age 20) are in a developmental stage of searching for identity, as described in The Search for Belonging [25]. When, as adults, they develop a mature and coherent identity, they can be powerfully motivated to live healthier lives [26], therefore, support for establishing a nursing professional identity during the second year may lead to improved health maintenance. In particular, counseling for the establishment of identity is expected not only to improve health, but also to enhance the ability to empathize, which is becoming increasingly important in the nursing profession, especially in today’s society where human relationships are becoming more and more tenuous. The establishment of identity in the second year is expected to enhance the ability to empathize [27], and for this reason, “building a foundation of identity” is necessary for second-year students.

On the other hand, the lifestyle habits of second-year students were also less favorable than those of first-year students, with increases in the rate of meal skipping and number of students sleeping less than six hours a night. Some college students try to lose weight through inappropriate methods, such as skipping meals [13], and the rapid spread of smartphones among young people has led to an increase in the number of students who suffer from lack of sleep [28]. Therefore, the strategy for second-year students should target “reflection on healthy lifestyle habits” in order to identify changeable lifestyle habits.

#### 4.2.3. Third-Year Students

The mean K10 score of third-year students (15.9) was highest among all the groups, and they also had the highest percentages of anxiety about studying (66 students, 89.2%) and clinical practice (67 students, 90.5%) compared to the other grades, a finding that differs from a report [10] that nursing students’ mental health scores improve as they progress to a higher year of college. In the third year, lectures, practical training, and clinical practice in specialized education become more challenging, and systematic education in various specialized areas of nursing generally reaches its climax. It has already been shown that higher academic burdens such as exams and assignments are associated with higher stress levels [10], especially in nursing education, where the level of difficulty of expertise and the volume of assignments may affect the student’s mental health status. Therefore, one possible reason why more students in their third year have anxiety about studies, clinical practice, and the future is that they are at a stage in their careers where competencies and aptitudes for the nursing profession are becoming more realistically demanded. It has been reported that nursing students experience “an unbearable reality,” “feeling the difference between learning and applying nursing care,” “disappointment at the diminished presence,” and “fear of becoming a nurse” in their clinical practice [29]. On the other hand, as will be discussed later, there is a trend toward improvement in the fourth-year students. Therefore, “strengthening the competencies and aptitude of third-year students for the nursing profession” is necessary by creating opportunities for fourth-year students to share their experiences about how they overcame the third-year challenges through peer support and by collaborating with nursing teachers and professionals from other disciplines.

Among third-year students, 32% were skipping meals, and 42% were sleeping less than six hours, which was a higher percentage than that seen for first- and fourth-year students. Most third-year students were not exercising and had more unfavorable lifestyle habits than the other groups; one in three students was skipping meals and sleeping less than six hours, possibly leading to a high-risk group for future development of life-style-related diseases. Lack of sleep can lead to poor academic performance [30] and can become the cause and result of poor mental health [31]. Therefore, measures for third-year students, who show significant changes from second-year students in lifestyle habits, should target “prevention of high-risk lifestyle habits”.

#### 4.2.4. Fourth-Year Students

The mean K10 score of the fourth-year students (14.6) was not significantly different from the other year groups. Additionally, about 80% of fourth-year students were anxious about clinical practice, although their level of anxiety was not as high as that of third-year students. This suggests that final year students in the bachelor’s program may develop better stress coping strategies by overcoming the challenges they faced in the third year [10]. In addition, about 80% of third-year students were anxious about their future, which was the highest percentage compared to other year groups. In fourth-year students, potential anxiety about choosing a post-graduation career path and preparing for national exams becomes apparent [32]. Fourth-year students are culminating their bachelor’s program and are at the stage of seriously confronting their future, and if their decision making is unclear, it may lead to early job turnover after employment [9]. Therefore, it will be necessary to provide “career support” for health care professionals as part of their vocational education, as well as to deepen their way of thinking as part of their liberal arts education in the bachelor’s degree program.

The percentage of students skipping meals increased as the year of college progressed, with fourth-year students showing the highest rate compared to other year groups. Skipping meals has been reported to correlate with stress, anxiety, depression, and significantly higher incidences of menstrual problems and concerns [33,34]. Despite their professional knowledge, nursing students did not engage in appropriate health behaviors to maintain their health. It is important, of course, that nursing students continue to adhere to their own health-promoting behaviors in their future careers (WHO, 2013) [35] because they are in a position to be models for health behaviors [13], and better health behaviors are related to higher quality of health care. Therefore, the strategy for fourth-year students should be aimed at “health development as lifelong health care professionals”. 

## 5. Conclusions

To enhance the future success of students in the bachelor’s program for nursing in Japan, the following strategies are recommended: “maintaining a healthy lifestyle” and “building a foundation for college life” for first-year students; “reflection on healthy life-style habits” and “building a foundation of identity” for second-year students; “prevention of high-risk lifestyle habits” and “strengthening the competencies and aptitudes for the nursing profession” for third-year students; and “health development as lifelong health care professionals” and “career support” for fourth-year students.

## 6. Limitation and Recommendation

This study has some limitations that need to be acknowledged. Firstly, this was a cross-sectional design and not a longitudinal study. In the future, longitudinal studies are needed to track changes in the nursing profession across the lifespan, as well as changes that occur as the years in nursing college progress. Secondly, no differentiation has been made between men and women, as it was determined that the sample sizes were too different to make comparisons. It would be interesting to see if there are psychological differences between these two groups in each of the college years. Thirdly, we targeted only nursing students as study subjects, but it would be interesting to compare school year groups for other health care professionals such as physicians, physiotherapists, etc. Finally, one important issue for future research is to perform assessments with not only subjective questionnaires, but to also measure objective variables such as physical activity and sleep duration using instruments such as accelerometers, and to measure biochemical variables such as hormones and cytokines.

## Figures and Tables

**Table 1:** Association between students of each year and demographic characteristics of students (N = 339).

	Year	1st		2nd		3rd		4th		Total				
Variable		Mean ± SD or n (%)		Mean ± SD or n (%)		Mean ± SD or n (%)		Mean ± SD or n (%)		Mean ± SD or n (%)		ANOVA or χ2	*p* Value	Tukey or z Test
K10 score avarage		13.1 ± 5.5		13.8 ± 6.7		15.9 ± 8.4		14.6 ± 5.7		14.3 ± 6.6		2.739	**	1st < 3rd *
Number of students		104	(100.0)	81	(100.0)	75	(100.0)	79	(100.0)	339	(100.0)			
Anxiety														
overall studies	Yes	60	(58.8)	53	(66.3)	66	(89.2)	47	(60.3)	226	(67.7)	21.343	***	1st, 2nd, 4th < 3rd *
	No	42	(41.2)	27	(33.8)	8	(10.8)	31	(39.7)	108	(32.3)			
clinical practice	Yes	36	(35.3)	57	(71.3)	67	(90.5)	63	(80.8)	223	(66.8)	72.000	***	1st < 2nd, 3rd, 4th *,2nd > 1st, 3rd *
	No	66	(64.7)	23	(28.8)	7	(9.5)	15	(19.2)	111	(33.2)			3rd > 1st, 2nd *, 4th > 1st *,1st < 4th *
future career path	Yes	62	(60.8)	54	(67.5)	57	(77.0)	62	(79.5)	235	(70.4)	9.491	*	1st < 4th *
	No	40	(39.2)	26	(32.5)	17	(23.0)	16	(20.5)	99	(29.6)			
life in general	Yes	32	(31.4)	27	(33.8)	18	(24.3)	14	(17.9)	91	(27.2)	6.304	†	
	No	70	(68.6)	53	(66.3)	56	(75.7)	64	(82.1)	243	(72.8)			
personal relationships	Yes	29	(28.4)	29	(36.3)	17	(23.0)	20	(25.6)	95	(28.4)	3.784	ns	
	No	73	(71.6)	51	(63.8)	57	(77.0)	58	(74.4)	239	(71.6)			

ANOVA: One-way analysis of variance; † 0.05 < *p* < 0.10, * *p* < 0.05, ** *p* < 0.01, *** *p* < 0.001.

**Table 2:** Association between students of each year and lifestyle of students (N = 339).

	Year	1st Mean ± SD or n (%)		2nd Mean ± SD or n (%)		3rd Mean ± SD or n (%)		4th Mean ± SD or n (%)		Total Mean ± SD or n (%)		χ2	*p* Value	z Test
Variable														
Lifestyle														
dietary habits	eating regularly	12	(11.8)	21	(26.3)	24	(32.4)	31	(39.7)	88	(26.3)	19.803	***	1st < 3rd, 4th *
	skipping meals	90	(88.2)	59	(73.8)	50	(67.6)	47	(60.3)	246	(73.7)			
sleep duration a night	less than six hours	21	(20.8)	18	(22.5)	31	(41.9)	15	(19.2)	85	(25.5)	13.628	**	1st,4th < 3rd *
	more than six hours	80	(79.2)	62	(77.5)	43	(58.1)	63	(80.8)	248	(74.5)			
exercise	don’t exercise regularly	83	(83.0)	64	(81.0)	73	(98.6)	61	(79.2)	281	(85.2)	14.241	**	1st,2nd,4th < 3rd *
	exercise regularly	17	(17.0)	15	(19.0)	1	(1.4)	16	(20.8)	49	(14.8)			

* *p* < 0.1, ** *p* < 0.01, *** *p* < 0.001.

## Data Availability

The data presented in this study are available on request from the corresponding author. The data are not publicly available due to the Ethical Guidelines for Epidemiological Research by the Japanese Government.
